# The impact of freeze-dried Baiyedancong-Oolong tea aqueous extract containing bioactive compounds on the activities of CYP450 enzymes, the transport capabilities of P-gp and OATs, and transcription levels in mice

**DOI:** 10.29219/fnr.v68.10605

**Published:** 2024-09-27

**Authors:** Miaogao Zhang, Zhenguo Qiu

**Affiliations:** 1 College of Light Industry and Food Sciences, Academy of Contemporary Agricultural Engineering Innovations, Zhongkai University of Agriculture and Engineering, Guangzhou, China

**Keywords:** Oolong tea, CYP450, P-gp, EGCG, caffeine, polyphenols

## Abstract

In this study, (−)-epigallocatechin gallate (EGCG) and caffeine extracted from freeze-dried autumn Baiyedancong Oolong tea (FBOT) were orally administered to mice for 7 consecutive days to explore the effects of BOT and its bioactive compounds on the activities and transcription levels of CYP450 enzymes, intestinal effluence transporter P-gp, and renal ingestion Organic Anion Transporters (OATs). Concurrently, EGCG and caffeine enhanced the activities of CYP3A, CYP2E1, and CYP2C37 in the liver of mice, while impairing the transport capabilities of P-gp and OATs. Reduced levels of MDR1 encoding P-gp transcription in the small intestine and renal OAT1 and OAT3 revealed that transcription was involved in the regulation of CYP450, P-gp, and OATs. The reduced transcription level of liver CYP2E1 suggested that CYP2E1 activity may have been elevated due to alternative mechanisms, but not through transcription. The absorption, metabolism, and excretion of drugs may be influenced by the daily consumption or high-dose administration of BOT and its related products, in which EGCG and caffeine may make great contributions.

## Popular scientific summary

Baiyedancong Oolong Tea (BOT) is a popular Chinese tea known for its health-promoting compounds, particularly ()-epigallocatechin gallate (EGCG) and caffeine. While these compounds offer benefits, they may also impact how the body processes medications. In this study, we investigated the effects of BOT and its key components on drug metabolism in mice. The mice were given BOT extract, EGCG, and caffeine over seven days. The study examined the activity of liver enzymes, such as cytochrome P450s (CYP450s), which are crucial for metabolizing drugs, and the function of drug transporters like P-glycoprotein (P-gp) and organic anion transporters (OATs), which help in drug absorption and excretion. The results showed that BOT, EGCG, and caffeine increased the activity of liver enzymes, suggesting they could accelerate drug metabolism, potentially reducing drug effectiveness. Conversely, these compounds also decreased the activity of P-gp and OATs, which could impair drug absorption and excretion, possibly leading to higher drug levels in the bloodstream and an increased risk of side effects. These findings highlight the need for caution when consuming BOT, especially for individuals taking medications, as it may affect drug efficacy and safety.

Guangdong typical Baiyedancong Oolong tea (BOT), a significant representative of Chinese Oolong tea, was designated by the Ministry of Agriculture’s Crop Variety Certification Committee as the national tea tree excellent seed in 2002 (1). It is distributed throughout Guangxi and Hainan in addition to Guangdong Province, where it has been promoted on an area of 200,000 acres. As a result of their extreme convenience, bottled or canned tea that is ready to consume has become an integral part of human life. Multivitamin tablets containing 50 to 630 mg of (−)-epigallocatechin gallate (EGCG) and caffeine per tablet are an example of the increased variety of health tea products available at present. Chronic diseases, including cancer, metabolic disorders, and cardiovascular disorders, are increasingly being treated with these products as pharmaceutical supplements (2).

However, high and/or prolonged doses of tea may be hazardous when consumed improperly. Ingestion of green tea polyphenol extract at a high dose has been linked to reports of liver poisoning (3). The long-term ingestion of old brick tea, which is rich in fluorine, has been a subject of concern among Chinese nomadic borderers since the 1980s. The interaction between tea and drugs has garnered considerable attention (4). Contemporary reports suggest that tea polyphenols (TPs) may exert diverse influences on drug bioavailability, encompassing direct binding with drugs, modulation of drug-metabolizing enzyme and transporter expression and activity, and consequent enhancement or inhibition of pharmacodynamic effects (5). Significant effects on drug metabolisms, metabolic enzymes, and transporter activities have been attributed to the ingestion of green tea extract or its primary components in high doses (6). Pharmacokinetics, therapeutic efficacy, and drug bioavailability may be influenced by alterations in blood drug concentration, metabolic enzyme and transporter activities, and daily dosage (7).

Interest has been greatly piqued regarding the impact of tea polyphenols on CYP450 and P-gp, given the critical role that these transporters play in drug metabolism. The activities and quantities of CYP450 enzymes can be influenced by drugs and other exogenous substances, which may result in metabolic drug interactions (8). So far, previous studies have explored the regulatory effects of green tea extract and EGCG. Misaka, Kawabe (9) conducted an *in vitro* study using human liver and small intestine as materials, demonstrating that green tea polyphenols simultaneously inhibited the activities of CYP450, CYP2B6, CYP2C8, CYP2C19, CYP2D6, and CYP3A, which was suggested that daily consumption of tea polyphenols could significantly inhibit CYP450 activity clinically. Another study (10) found that although Japanese green tea extract had no obvious effect on the activities of CYP3A and CYP1A2 in mice, it significantly improved CYP1A1 activity. In addition to green tea polyphenols, other components in tea may also affect drug metabolism. A study based on metabolic kinetics from Belayneh and Molla (11) showed that caffeine accelerated the metabolism of promethazine in healthy individuals, which might be responsible for the enhancement of CYP2D6 activity by caffeine.

Previous studies have also shown that tea polyphenols could reduce P-gp transport capacity and inhibit its gene expression. Mei, Qian (12) observed that tea polyphenols (40 μg/mL) and EGCG (10 μg/mL) treated KB-A1 cells suppressed ATPase activity, inhibiting P-gp activity. Choi and Burm (13) reported that EGCG and EGC reduced P-gp expression in rat small intestine. However, inconsistent findings have also been reported in some studies. A study from Knop, Misaka (14) indicated that EGCG significantly increased the expression of P-gp in intestinal epithelial cells. Simultaneously, flow cytometry results showed that EGCG obviously improves the relative fluorescence intensity of cells, which was mutually verified with a western-blotting assay. *In vitro* studies have also revealed that different catechin monomers may have opposite effects on P-gp at high and low doses (15).

In contrast, prior studies have placed less emphasis on the impact of Chinese characteristic Oolong tea (e.g. BOT) on drug-metabolizing enzymes and transporters. In contrast to green tea, Oolong tea possesses a distinct amalgamation of bioactive constituents and consumption ways. Despite pertaining to the identical variety of Oolong tea, variations in processing techniques may result in distinct ingredient compositions and contents, thereby potentially influencing the *in vivo* effects of drug metabolisms (16).

In our preliminary study, spring, summer, autumn, and winter BOT were individually dried using freeze- and conventional hot air-drying methods. Subsequently, a series of sensory, quality and physicochemical assessments were carried out to select the BOT that was satisfied with consumption. The autumn freeze-dried BOT (FBOT) was employed in this study according to the results of our preliminary study. Herein, oral administration of FBOT and its primary bioactive compounds, including BOT polyphenols, EGCG, and caffeine, to mice for 7 days at both high and low doses was performed in order to investigate the impact of these compounds on the activities of CYP450 enzymes and transcription expressions in the liver and intestine of the mice. Additionally, the transcription levels and transport capacities of the small intestinal transporter P-gp and the renal transporter OATs were assessed in relation to the effects. Theoretical underpinnings for the appropriate administration of BOT and its related products during medication could be established by our findings.

## Materials and methods

### Materials

The initial stages of BOT production included the following: leaf harvesting, leaf cooling, leaf rolling, leaf drying, fine manipulation, and leaf frying. Tea samples underwent a freeze-drying process after being rolled and divided into six portions. A temperature of −20°C was maintained prior to freezing in the freeze-drying process. Zili Chromatography Co., LTD. supplied the chromatographic pure methanol and acetonitrile that were acquired (Guangzhou, China). The NADH^+^ system was acquired from Sigma (St. Louis, MO, USA). Amphenoxine, Genopsin Chemical Reagents Co., LTD. (Shanghai, China), supplied the erythromycin, while Genview (Houston, TX, USA) supplied the aminobiline.

### Preparation of FBOT aqueous extract and extraction of tea polyphenols, EGCG, and caffeine

In preparation for further analysis, freeze-dried autumn BOT (FBOT) was ground into tea powders before being filtered through an 80-mesh sieve. For 10 min, the tea powders (4.375 g) were extracted using 300 mL of boiling ddH_2_O. Following the collection of the extract, the residue underwent two additional washes with 100 mL of boiling ddH_2_O. The resulting liquid solution was then combined with the extracted sample and utilized as a stocking extract. To obtain a high-concentration extract, this stocking extract was purified under reduced pressure at 65°C. Following this, the final BOT working extracts were obtained by diluting the high-concentration extract five times. Our preliminary study yielded high and low concentrations of FBOT (36.59 and 7.32 mg/mL, respectively).

### Extraction of tea polyphenol

Fifty grams of tea leaves was extracted for 4 h at room temperature using 400 mL of 75% ethanol. The leaves underwent a double ethanol extraction process after being separated. At 40°C, the ethanol was evaporated in a rotary evaporator under reduced pressure. To eliminate caffeine, the dried extract was suspended in 500 mL of distilled water and extracted with dichloromethane. After separating the decaffeinated water solution of tea extract through a XAD-16 resin column, it was eluted with 100% ethanol and rinsed with five volumes of distilled water. By utilizing a rotary evaporator, the extract was dried.

### Gallic acid equivalent

The experiment was conducted in accordance with a previous report (17), utilizing the Folin-Ciocalteau reagent. A ThermoMax microplate reader was utilized to measure the absorbance at 755 nm (Molecular Devices). Using the standard curves, the average absorbance of each sample was converted to mg/g gallic acid equivalent.

### HPLC condition for the analysis of catechins and caffeine

Caffeine and catechins were analyzed utilizing a Water Alliance 2695 HPLC system integration with a PDA detector. EGCG and caffeine were separated using an Agilent Zorbax SB C18 column in conjunction with a 0.4% phosphoric acid in water and acetonitrile gradient. A wavelength of 280 nm was utilized.

### Animal treatment and groups

CYP450 enzyme activities and P-gp transport capacity determination and treatment

The Laboratory Animal Center of Guangzhou University of Traditional Chinese Medicine generously supplied 144 SPF-grade adult mice (mean 5.0 ± 5.0 g, 72 male and 72 female) (certificate number: SCXK 2013-0020). The mice were maintained on a 12-h light and dark cycle and were provided with a standard diet and endless water. Experimental procedures and animal welfare were conducted in accordance with institutional guidelines for animal care. For 7 consecutive days, mice were gavaged twice daily at 8:00 am and 6:00 pm at a rate of 20 mL/kg. The mice were allocated into 12 groups (*n* = 12) in a random manner: control (ddH_2_0); carboxymethylcellulose (CMC, 0.5%, v/v); rifampicin (40 mg/kg; supplied by Guangdong Huanan Pharmaceutical Group Co. LTD, Guangzhou, China); ketoconazole (1.8 mg/kg; sourced from Nanjing Baijingyu Pharmaceutical Co. LTD, Nanjing, China); high and low dose of FBOT (FBOT-H and FBOT-L, 1463.7 and 292.74 mg/kg/d, respectively), high and low dose of BOT polyphenols (BOTP-H and BOTP-L, 901.6 and 180.32 mg/kg/d, respectively), high and low dose of EGCG extracted from BOT (EGCG-H and EGCG-L, 531.72 and 106.34 mg/kg/d, respectively), and high and low dose of caffeine extracted from BOT (caffeine-H and caffeine-L, 108.85 and 21.77 mg/kg/d, respectively). Abstention sulfur, rifampicin, and ketoconazole were all dissolved in 0.5% CMC. On the morning of the 8th day, every subject underwent gavage. All mice were gavaged acetaminophen (APAP, 20 mg/kg) at 10 mL/kg after 60 min.

OATs transport capacity determination and treatment

The Laboratory Animal Center of Guangzhou University of Traditional Chinese Medicine supplied a cohort of 132 adult mice of SPF-grade NIH (mean 5.0 ± 5.0 g, 66 male and 66 female). The feeding conditions persisted were the same as indicated above. Mutagens were gavaged twice daily at 8:00 am and 6:00 pm for 7 days at a rate of 20 mL/kg. At random, 11 groups (*n* = 12) of mice were formed, including control (ddH_2_0), CMC (0.5%, v/v), probenecid (50 mg/kg, Sigma, MO, USA), FBOT-H and FBOT-L (1463.7 and 292.74 mg/kg/d, respectively), BOTP-H and BOTP-L (901.6 and 180.32 mg/kg/d, respectively), EGCG-H and EGCG-L (531.72 and 106.34 mg/kg/d, respectively), and caffeine-H and caffeine-L (108.85 and 21.77 mg/kg/d, respectively). Probenecid was dissolved in 0.5% CMC. On the 8th day, each subject was gavaged in the morning.

### Determination of serum APAP concentration

The methodology proposed by Alonso, James, Zhang, Squires, and Group (2015) is cited. Following 60 min of APAP administration, blood samples were obtained from the severed heads of mice and placed in dry centrifuge tubes. The samples were centrifuged at 2,797.5 × g, 4°C for 5 min, following a 30-min period of standing. A supernatant was obtained from which 1 mL of trichloroacetic acid was thoroughly combined with 0.25 mL of serum. Following a 5-min centrifugation at 10,000 × g, 4°C, 1 mL of supernatant was obtained and combined with 0.25 mL of hydrochloric acid at a concentration of 6 mol/L and 0.25 mL of a 20% NaNO_2_ solution. After allowing the mixture to rest for 5 min to allow for a complete reaction, 0.5 mL of a 15% sulfionic acid solution was added, while ultrasound was used to ensure that no bubbles formed. Following the addition of 1 mL of a 20% NaOH solution, the mixture was left at room temperature for 20 min. At 430 nanometers, absorbance was measured. The control was the mixture that did not contain APAP. The inter-day precision was 2.78 to 3.58%, while the within-day precision was 1.35 to 2.12%, when the linear range of APAP was between 0.5 and 50 ug/mL. Reproducibility ranged between 98 and 113%.

### Extraction of liver and intestinal microsomes

The method of extraction utilized to separate liver and intestinal microsomes (LM and IM, respectively) was calcium precipitation differential centrifugation as described in Hatley, Jones (18). Phosphate-balanced solution (PBS, pH = 7.4) was utilized to extract the liver and small intestine at 0°C through a tissue homogenizer operating at a speed of 142 × g for 5 cycles, for a total of 30 s. The mixture was subsequently centrifuged in succession at 9,000 × g for 20 min, 50,000 × g for 30 min, and 50,000 × g for 30 min. Following collection, the precipitates were combined with an equivalent volume of PBS solution. At −80°C, the LM and IM were preserved. The Bradford protein concentration assay kit was utilized to ascertain the protein concentration of LM and IM (Jiemei Gene Medicine Technology Co., LTD, Shanghai, China).

### Determination of CYP3A activity in mice liver and intestine

The 0.5 mL samples, which contained 1 mg/mL protein, were combined in a 37°C water bath for 3 min with 0.3 mL of 0.02 mol/L HEPES buffer (pH = 7.4, Dingguo Changsheng Biotechnology Co., LTD, Beijing, China) and 0.1 mL of erythromycin or aminopyrine at a concentration of 86.4 mmol/L. A system for generating NADPH was introduced into the mixture (2 U/mL G-6-PDH, 20 mmol/L NADP^+^, 20 mmol/L G-6-P, and 20 mmol/L MgCl_2_) and incubated at 37°C for 60 min. Following the addition of 0.5 mL of 30% trichloroacetic acid to terminate the reaction, 20,000 × g of acetic acid at 0°C for 10 min was centrifuged. After combining 1.0 mL of supernatant with 1 mL of NASH reagent in a water bath at 60°C for 10 min, the mixture was allowed to stand at room temperature for an additional 20 min. At a wavelength of 412 nm, the absorbance was measured (19). Units of nmol formaldehyde per milligram of protein per minute constituted the enzyme activity. For the determination of liver CYP3A activity in mice using erythromycin, aminopyrazoline, and erythromycin as substrates, the standard curves for formaldehyde were as follows: *Y* = 0.1945X + 0.0157, *r* = 0.9988, *n* = 5 (linear range: 1.5–15 nmol); *Y* = 0.1947X + 0.0156, *r* = 0.9988, *n* = 5 (linear range: 0.02–0.4 nmol); and *Y* = 0.1945X + 0.0157, *r* = 0.9988, *n* = 5 (linear range: 0.05–0.5 nmol), respectively.

### Determination of liver CYP2E1 activity in mice

A 0.05 mL of 0.1 mol/L aniline solution was combined with 0.9 mL of LM suspension in a water bath set at 37°C for 3 min. The mixture was agitated for an additional 3 min after a 0.05 mL solution of hydroxyl isopropyl benzene peroxide was introduced. After incorporating 0.4 mL of 35% trichloroacetic acid, the sample was centrifuged at 20,000 × g, 0°C for 10 min; 0.5 mL of supernatant was combined with a sodium carbonate solution with a concentration of 1 mol/L. The solution was then treated for 30 min at room temperature with 0.5 mL of a 2% phenol reagent. At 630 nm, the absorbance was measured (20). The p-aminophenol content was determined and recorded. nmol p-aminophenol mg protein-1 min-1 represented the CYP2E1 activity. *Y* = 0.005X – 0.087, *R* = 0.997, and *n* = 5 comprised the obtained standard curve (linear range: 12.5–125 nmol).

### Determination of intestinal CYP2C37 activity in mice

Specific parameters for chromatographic columns: flow rate: 1.00 mL/min; column temperature: 30°C; detection wavelength: 280 nm; and the mobile phase consisted of acetonitrile-0.01 mol/L ammonium acetate (48:52 (v/v), pH = 3.6). The Agilent ZORBAX SB-C18 reversed-phase column (150 mm × 4.6 mm, 5 μm) was utilized. The enzyme reaction system is comprised of 20 μL of 80 mol/L diclofenac solution, 50 μL of 1 mg/mL LM protein solution, 30 μL of NADPH generation system, and 80 μL of potassium phosphate buffer (pH = 7.4). Prior to initiating the reaction, 20 μL of β-NADP^+^ was introduced into the enzyme system, which had been pre-incubated in a 37°C water bath for 3 min. Following a 15-min incubation period, 180 μL of acetonitrile was introduced to terminate the reaction. After adding 20 μL of an internal standard solution, the sample underwent a 15-min centrifugation at 12,000 g at 4°C. A total of six supernatant samples (20 μL) were gathered in preparation for HPLC analysis. Gradient doses of 0.5, 1, 2.5, 5, 7.5, 10, 15, and 20 μmol/L of the 4-hydroxydiclofenac standard enzyme incubation system were analyzed via HPLC. The dose of 4-hydroxydiclofenac was represented by the horizontal coordinate of the standard curve (C (μmol/L, X)), whereas the peak area ratio of 4-hydroxydiclofenac to the internal standard (A1/A2, Y) comprised the vertical coordinate. The standard curve *Y* = −0.7618X + 1.8178 was obtained using equation, with *R* = 0.9997 and *n* = 5 (linear range: 0.5–20 μmol/L). The detection limit (LOD) for five parallel measurements was 0.25 mol/L (S/N ≥ 3, *n* = 5), and the RSD was below 1.15% (21).

### Extraction of total RNA

The RNA extraction kit was utilized to extract total RNA from the liver, small intestine, and left kidney of mice in accordance with its instructions (TaKaRa, Japan). The absorbance at 260 and 280 nm was determined using ultraviolet spectrophotometry. For real-time quantitative PCR (RT-PCR) analysis, samples were occupied within the A260nm/A280nm range of 1.8–2.2.

### Determination of mRNA expressions of liver and intestinal CYP3A11, liver CYP2E1, liver CYP2C37, intestinal MDR1, renal OAT1 and OAT3 in mice

In accordance with the guidelines provided in the PrimeScriptTM RT assay kit (Takara, Tokyo, Japan), reverse transcription was executed utilizing ABI7500 real-time fluorescent quantitative PCR equipment. The reaction conditions comprised one cycle at 85°C for 31 s and 42°C for 15 min. The reaction mixture comprised 2 μL of total RNA, 2.5 μL of PrimeScript®Buffer (5x), 1 μL of Oligo dT Primer at 50 μmol/L, 3 μL of Random 6mers at 100 μmol/L, and an additional 20 μL of ddH_2_O (without RNase). A 20 μL system was utilized to conduct the PCR, employing the SYBR®Premix Ex TaqTMIII assay kit (Takara, Tokyo, Japan). The system contained the following components: 10 μL of SYBR® Premix Ex TaqTM⅏, 0.8 μL each of forward and reverse primers (Shanghai Sangong Biological Engineering Co., LTD, Shanghai, China), 0.4 μL of ROX Reference Dye, 2 μL of cDNA sample, and 20 μL of DEPC water. Reactions for PCR were as follows: 30 s of pre-degeneration at 95°C was followed by 40 cycles of the cycle stage, which consisted of 15 s of denatured 95°C, 30 s of annealing at 56°C, and 31 s of extension at 72°C. GAPDH was utilized as an internal citation, and 2^-ΔΔCt^ was utilized to determine the relative expression levels of the target genes. Table 1 details the prime sequences.

**Table 1:** Prime sequences for RT-PCR

Gene	Forward prime(5’ →3’)	Reverse prime(5’ →3’)
CYP3A11	CTCAATGGTGTGTATATCCCC	CCGATGTTCTTAGACACTGCC
CYP2E1	CACCGTTGCCTTGCTTGTCTG	CTCATGAGCTCCAGACACTTC
CYP2C37	CTGCATGACAGCACGGAGTT	GTGGCCAGGGTCAAATTTCTC
MDR1	CCCATCATTGCAATAGCAGG	GTTCAAACTTCTGCTCCTGA
OAT1	ATGCCTATCCACACCCGTGC	GGCAAAGCTAGTGGCAAACC
OAT3	CAGTCTTCATGGCAGGTATA	CTGTAGCCAGCGCCACTGAG
GAPDH	GGTGAAGGTCGGTGTGAACG	CTCGCTCCTGGAAGATGGTG-

### Determination of transporter P-gp-coupled ATPase activity

The activity of the transporter P-gp-coupled ATPase was assessed in accordance with the ultramicro ATPase kit’s instructions (Abcam, Cambridge, FC, USA).

### In vitro PAH uptake in renal sections

One hour following the final gavage, the mice were sacrificed. Both sides of the kidney were immediately removed, while the right kidney was stored at −80°C for mRNA determination. The left kidney, on average, was divided into two sections along its long axis from the renal hilum. Each section was then divided equally into three strips along its long axis and further divided into three pieces. The kidney was placed in a 12-well culture plate containing 1 mL of PBS supplemented with adequate oxygen after rinsing it with cold PBS and drying it with filter paper. A well containing 2 mmol/L PAH was used to incubate a kidney slice for 20 min at 37°C with 5% CO_2_. The well was agitated for 5 s every 5 min. In order to halt the reaction, 250 μL of a 10% trichloroacetic acid solution was introduced. Five times, for a total of ten s at each interval, the incubated renal tissues were homogenized with phosphoric acid buffer with a pH of 7.4. The protein concentration was ascertained using the Folin phenol method following centrifugation at 2,000 × g for 30 min, from which the supernatant was collected. According to a prior investigation, the PAH in the supernatant was ascertained (22). The PAH standard curve obtained is *y* = 0.3937x – 0.0025, *R*^2^ = 0.9986, *n* = 10.

### Statistical analysis

The statistical software SPSS13.0 was utilized. The findings were presented in the form of means with standard errors (SE), and to examine the variations between groups, an inter-group *T*-test was performed. *P*-values less than or equal to 0.05 and 0.01 were deemed to be statistically significant.

## Results

### The main components of FBOT

Tea aqueous extract encompasses all tea components soluble in hot water, such as amino acids, polyphenols, caffeine, soluble sugar, and soluble protein. Its composition could be indicative of the quality of the tea. Tea that is rich in tea aqueous extract invariably possesses a robust flavor. Our preliminary study utilized BOT sourced from various seasons and subjected to distinct processing methods (vacuum freeze-drying, vacuum drying, and heat-drying) to assess and contrast their sensory attributes, aqueous extract concentrations, and principal bioactive constituents (data were not shown). The findings presented in Table 2 indicate that the autumn FBOT obtained the highest sensory evaluation score, and its tea aqueous extract content (41.82%) was significantly greater than that of the other BOT. Tea polyphenols constitute a significant proportion (18–36%) of the dry weight of tea and are a primary contributor to the bitter flavor observed in tea. The FBOT harvested in autumn contained the highest concentration of TP (25.76%) among autumn BOT processed using various techniques. A proportion of 3.47% of autumn FBOT is composed of amino acids, which are essential components of tea and contribute significantly to the flavor and freshness of tea soup. Conversely, autumn FBOT contained 3.12% caffeine. Caffeine is a significant bitter constituent found in tea, and its characteristics remain comparatively consistent. The freeze-drying process diminishes the impact of caffeine. Oolong tea contains four primary monomers of catechin, namely, EGCG, EGC, ECG, and EC. The autumn FBOT contained the highest percentages of EGCG, ECG, EGC, and EC (151.92, 32.35, 19.99, and 5.12%, respectively) of any autumn BOT processed using a variety of drying techniques. This finding suggested that vacuum freeze-drying may be a more effective method for preserving EGCG, ECG, EGC, and EC. The autumn FBOT extract, TP, EGCG, and caffeine were utilized for subsequent enzyme activities and mRNA expression analysis in accordance with the findings of our preliminary study.

**Table 2:** The main components in FBOT

Components	Content (%)
Aqueous extract	41.82 ± 1.54
Polyphenols	25.76 ± 1.17
Amino acids	3.47 ± 0.07
Caffeine	3.12 ± 0.07
EGCG	151.92 ± 2.35
ECG	32.35 ± 1.04
EGC	19.99 ± 0.07
EC	5.12 ± 0.07
Sum of catechin	209.38 ± 2.2

Values are mean ± standard deviation (mean ± SD, *n* = 3).

### The effects of FBOT extract and its bioactive compounds on CYP3A activity and mRNA expression in mice liver

**Fig. 1:**
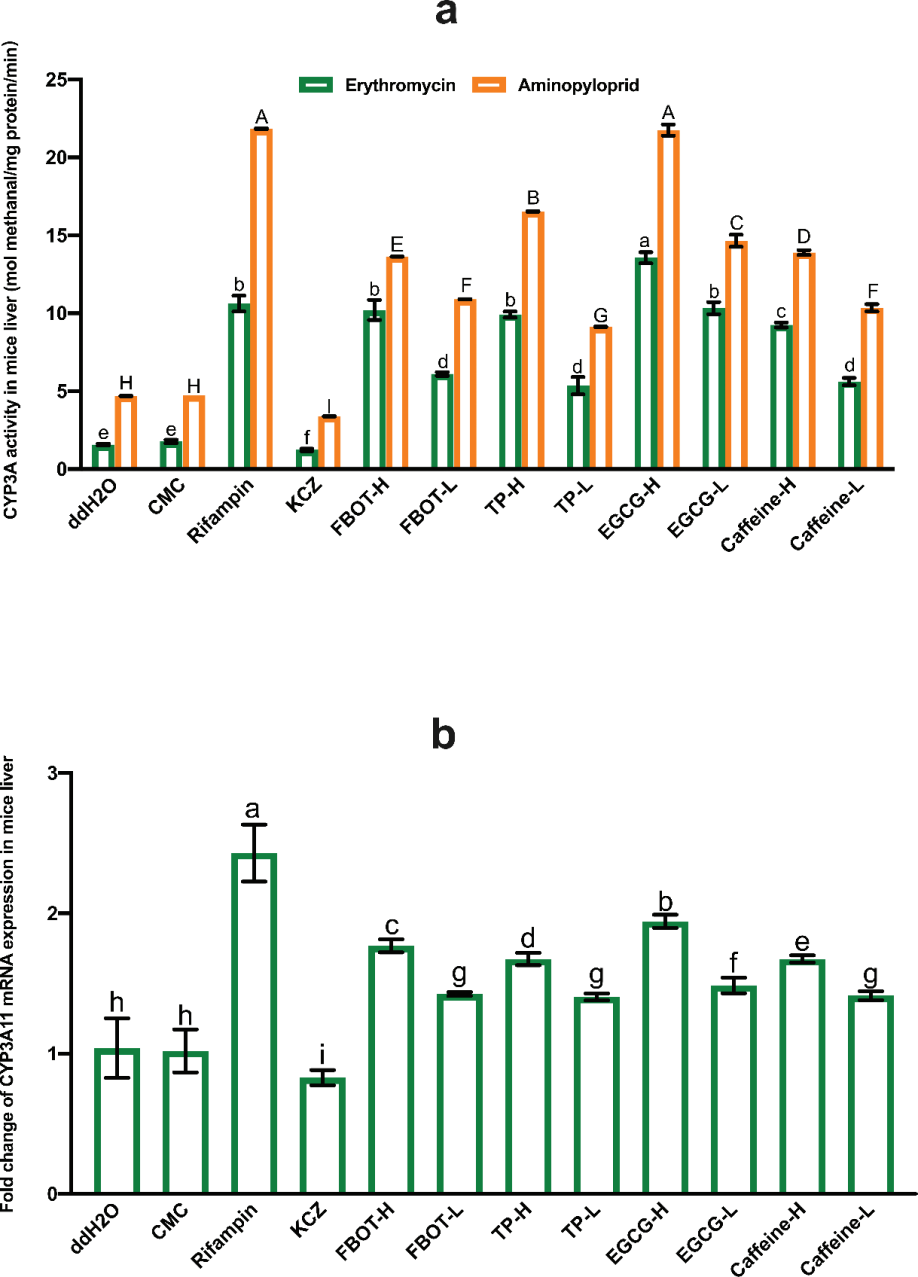
The activities of CYP3A (a) and mRNA expression of CYP3A11 (b) in mice liver. Values are mean ± SD (*n* = 3). Bars with uppercase letters (aminopyrine as substrate) and lowercase letters (erythromycin as substrate) represent a significant difference between each group (*P* < 0.05). FBOT-L and FBOT-H mean low and high dose of freeze-drying Baiyedancong Oolong tea, respectively; TP-H and TP-L mean high and low dose of tea polyphenols in BOT, respectively; EGCG-H and EGCG-L mean high and low dose of Epigallocatechin-3-gallate (EGCG) in BOT, respectively; caffeine-H and caffeine-L mean high and low dose of caffeine in BOT, respectively.

Among the essential isoenzymes involved in the metabolism of clinical drugs is CYP3A. In this study, CYP3A activity was assessed using two distinct substrates, erythromycin and aminopyloprid, separately. The obtained results were consistent with those illustrated in Fig. 1a. CYP3A activity did not differ significantly between the CMC and control groups (*P* > 0.05), indicating that CMC had no effect on CYP3A activity. The CYP3A activity of the mice induced with rifampicin was 6.4 times greater than that of the control group (*P* < 0.01). Conversely, the mice treated with ketoconazole exhibited a significantly lower CYP3A activity compared to the control group (78% of the control group). The CYP3A activity of mice exposed to FBOT, FBOTP, EGCG, and caffeine obviously increased in a dose-dependent manner when compared to the control group. Notably, the CYP3A activity of mice treated with EGCG-H reached the same level as that induced by rifampicin. Therefore, the induction of liver CYP3A activity of FBOT extract was strongly related to the polyphenols, EGCG, and caffeine. However, the induction of EGCG and caffeine on the CYP3A activity was not carried out in an accumulative way, but in other ways such as competition or independent induction.

The RT-PCR assay was used to determine the CYP3A11 mRNA level in the liver of mice to confirm whether the increased CYP3A activity in the liver is due to the transcription of the CYP3A11 encoding gene. The findings are illustrated in Fig. 1b. The findings indicated that the mRNA expression of CYP3A11 did not differ significantly between the control and CMC groups (*P* > 0.05). Rifampicin induced a substantial increase in the mRNA expression of CYP3A11 in the liver of mice, 2.24 times that of the control group (*P* < 0.05). In contrast, ketoconazole reduced this expression by a mere 70% in the control group. The liver CYP3A11 mRNA expression increased dramatically and dose-dependently in mice treated with high and low doses of FBOT, TP, EGCG, and caffeine, by 1.63, 1.27, 1.56, 1.31, 1.84, 1.35, 1.54, and 1.26 times, respectively, compared to the control group.

Collectively, this increased liver CYP3A11 mRNA expression in mice is consistent with its CYP3A activity in mice liver. However, the difference in CYP3A11 mRNA expression among treatment groups was lower than that of CYP3A activity among groups, suggesting that CYP3A activity in the liver may be regulated by variables other than CYP3A11 mRNA expression, including post-translation modification, translation level enhancement, and enzyme quantity stabilization.

### The effects of FBOT extract and its bioactive compounds on CYP2E1 activity and mRNA expression in mice liver

**Fig. 2:**
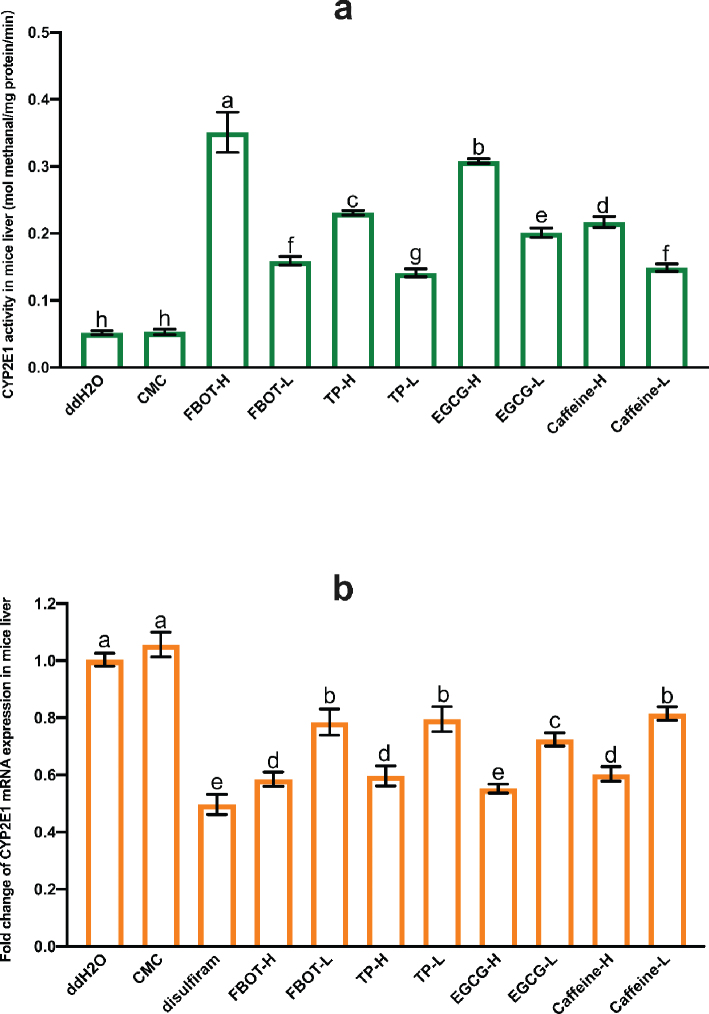
The activities of CYP2E1 (a) and mRNA expression of CYP2E1 (b) in mice liver. Values are mean ± SD (*n* = 3). Bars with different letters represent a significant difference between each group (*P* < 0.05). FBOT-L and FBOT-H mean low and high dose of freeze-drying Baiyedancong Oolong tea, respectively; TP-H and TP-L mean high and low dose of tea polyphenols in BOT, respectively; EGCG-H and EGCG-L mean high and low dose of Epigallocatechin-3-gallate (EGCG) in BOT, respectively; caffeine-H and caffeine-L mean high and low dose of caffeine in BOT, respectively.

Tea polyphenols, EGCG, and caffeine were selected to confirm the key bioactive compounds in FBOT extract that could regulate the CYP2E1 activity in mice liver. The results are shown in Fig. 2a. There was no significant change in CYP2E1 activity observed in CMC-treated mice when compared to the control group. The CYP2E1 activities of mice treated with FBOT, TP, EGCG, and caffeine were significantly higher than those of the control group (*P* < 0.05). In mice treated with high doses of FBOT, TP, EGCG, and caffeine, CYP2E1 activity was 5.89, 4.49, 5.97, and 4.21 times that of the control group, respectively. In mice treated with low doses of FBOT, TP, EGCG, and caffeine, CYP2E1 activity was 2.46, 2.72, 3.89, and 2.87 times that of the control group. Furthermore, the CYP2E1 activity in the livers of mice treated with FBOT and EGCG was significantly greater than that of mice treated with TP and caffeine.

Figure 2b illustrates the mRNA expression levels of CYP2E1 in the livers of mice that were subjected to various treatments. In general, there was no significant difference in the CYP2E1 mRNA levels between the control and CMC groups (*P* > 0.05). In comparison to the control group, CYP2E1 mRNA expression was halved in the absence of sulfur. In a similar fashion, the expression of CYP2E1 mRNA was significantly reduced (*P* < 0.05) in mice that were administered high and low doses of FBOT, TP, EGCG, and caffeine, by 62, 77, 56, 76, 47, 72, 46, and 76% of the control group, respectively. Furthermore, a notable disparity in mRNA expression was observed between treatments administered at high and low doses. Nevertheless, the level of CYP2E1 mRNA expression did not correspond to the observed CYP2E1 activity. CYP2E1 activity increased while mRNA expression and transcription were significantly downregulated in response to FBOT, TP, EGCG, and caffeine treatment. This suggested that the upregulated CYP2E1 activity was not accountable for the downregulated mRNA expression, but rather could be attributed to factors such as the quantity of stable enzyme.

### The effects of FBOT extract and its bioactive compounds on CYP2C37 activity and mRNA expression in mice liver

**Fig. 3:**
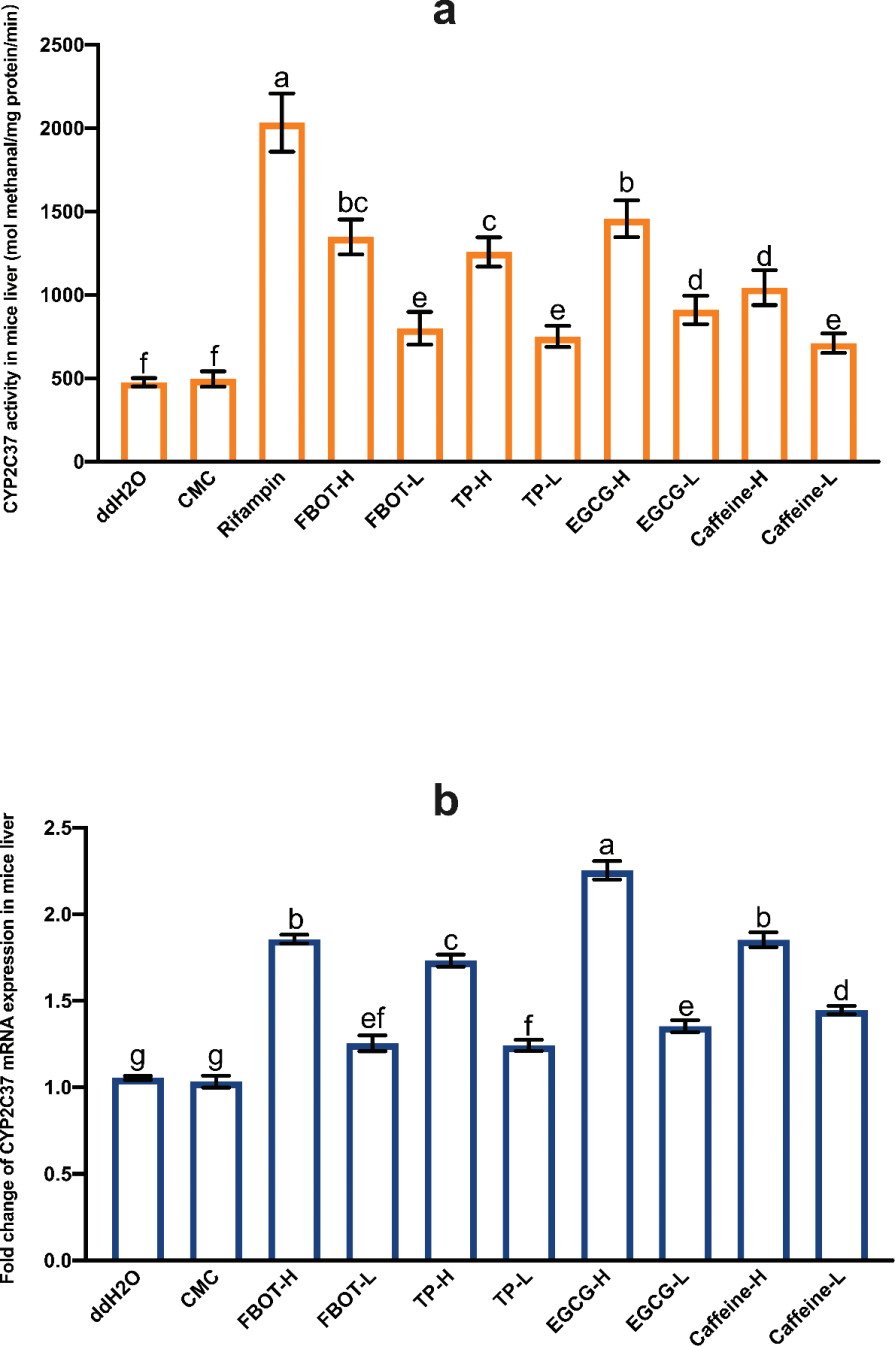
The activities of CYP2C37 (a) and mRNA expression of CYP2C37 (b) in mice liver. Values are mean ± SD (*n* = 3). Bars with different letters represent a significant difference between each group (*P* < 0.05). FBOT-L and FBOT-H mean low and high dose of freeze-drying Baiyedancong Oolong tea, respectively; TP-H and TP-L mean high and low dose of tea polyphenols in BOT, respectively; EGCG-H and EGCG-L mean high and low dose of Epigallocatechin-3-gallate (EGCG) in BOT, respectively; caffeine-H and caffeine-L mean high and low dose of caffeine in BOT, respectively.

As shown in Fig. 3a, the CYP2C37 activity of mice subjected to various treatments varied. The lack of a significant change in CYP2C37 activity among CMC-treated mice relative to the control group indicates that CMC had no effect on CYP2C37 activity. The activity of liver CYP2C37 in mice was found to be significantly increased by rifampicin, with a 4.59-fold increase compared to the control group (*P* < 0.05). Caffeine, FBOT, TP, and EGCG all dose-dependently increased CYP2C37 activity in the liver of mice. EGCG demonstrated the most pronounced induction effect. This effect, nonetheless, was less pronounced than that of rifampicin. In mice treated with low doses of FBOT, TP, EGCG, and caffeine, CYP2C37 activity was 1.75, 1.63, 1.94, and 1.52 times that of the control group, respectively. In the high dose of FBOT, TP, EGCG, and caffeine groups, CYP2C37 activity was 2.83, 2.71, 3.21, and 2.24 times that of the control group.

The lack of a statistically significant difference in CYP2C37 mRNA expression levels between the control and CMC groups (Fig. 3b) suggests that CMC had no effect on CYP2C37 mRNA levels. The mRNA expression of CYP2C37 in the liver was increased by rifampicin to a magnitude of 2.23 times that of the control group. In mice that were exposed to FBOT, TP, EGCG, and caffeine at both high and low doses, this expression was also increased (*P* < 0.05). Specifically, it was 1.77, 1.23, 1.72, 1.27, 1.79, 1.29, 2.07, and 1.37 times greater than that of the control group. Moreover, there was an extremely significant difference between the low and high doses. In contrast, the mRNA expression of CYP2C37 was greater in the group of mice treated with caffeine compared to the EGCG group, whereas its corresponding enzyme activity was lower in the EGCG group. This indicated that EGCG and caffeine may regulate CYP2C37 activity via mechanisms other than the regulation of CYP2C37 mRNA expression.

### The effects of FBOT extract and its bioactive compounds on CYP3A activity and CYP3A11 mRNA expression in mice intestine

**Fig. 4:**
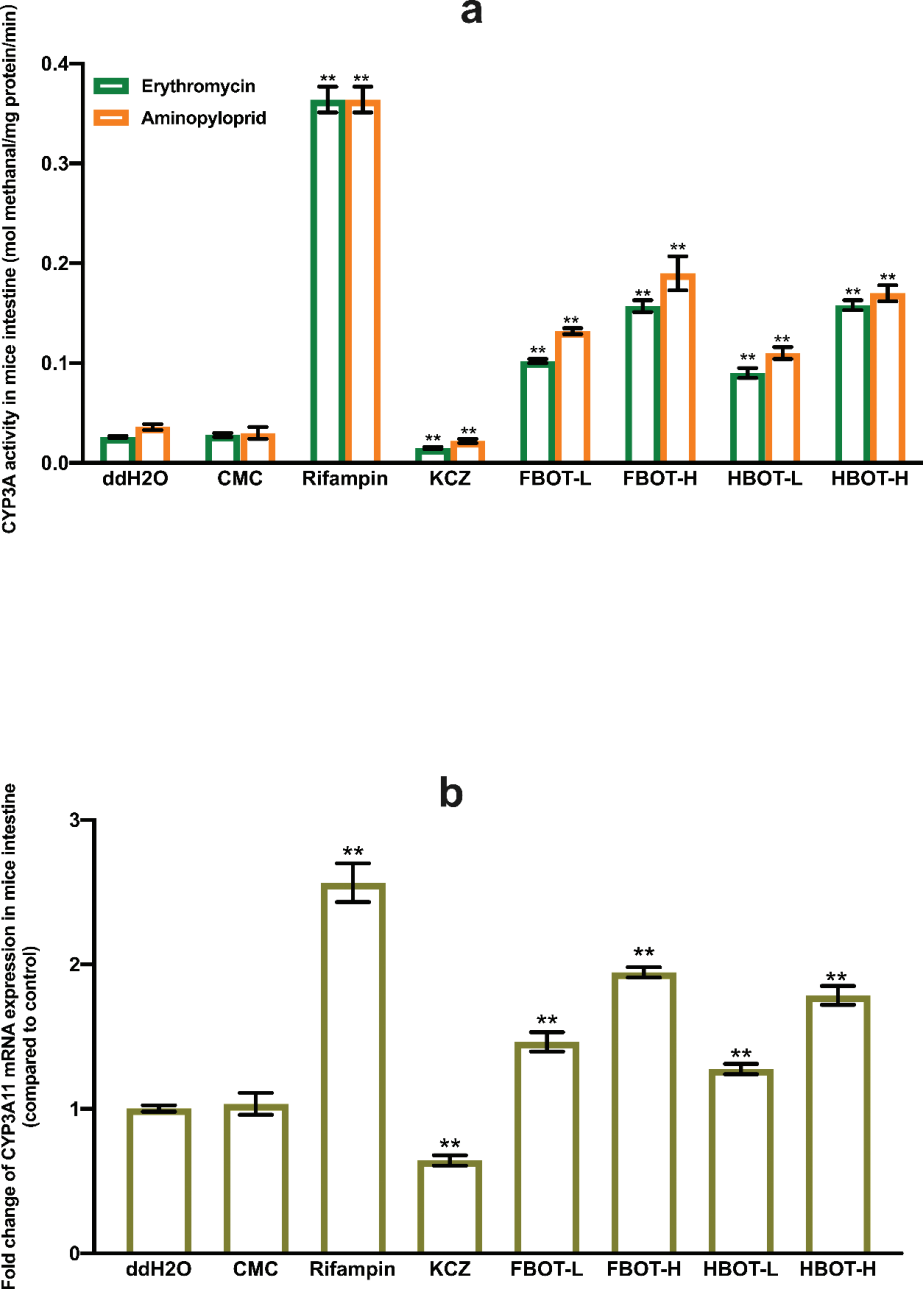
The activities of CYP3A (a) and mRNA expression of CYP3A11 (b) in mice intestine. Values are mean ± SD (*n* = 3). Bars with uppercase letters (aminopyrine as substrate) and lowercase letters (erythromycin as substrate) represent a significant difference between each group (*P* < 0.05). FBOT-L and FBOT-H mean low and high dose of freeze-drying Baiyedancong Oolong tea, respectively; TP-H and TP-L mean high and low dose of tea polyphenols in BOT, respectively; EGCG-H and EGCG-L mean high and low dose of Epigallocatechin-3-gallate (EGCG) in BOT, respectively; caffeine-H and caffeine-L mean high and low dose of caffeine in BOT, respectively.

The CYP3A activity in the intestinal tract of mice fed erythromycin was found to be comparable to that of aminopyriprid, as shown in Fig. 4a. The CYP3A activity was not influenced by CMC. However, rifampicin caused a substantial increase in CYP3A activity, surpassing that of the control group by 13.84 times (*P* < 0.01). Clearly, ketoconazole decreased CYP3A activity in the intestinal tract of mice by a mere 55% compared to the control group. The administration of FBOT, TP, EGCG, and caffeine at both high and low doses resulted in a dose-dependent enhancement of intestinal CYP2C37 activity in the liver. Among these substances, EGCG demonstrated the most pronounced induction effect. This effect, nonetheless, was less pronounced than that of rifampicin. The intestinal CYP2C37 activity of mice treated with high doses of FBOT, TP, EGCG, and caffeine was 5.89, 6.29, 8.59, and 5.86 times that of the control group, respectively. In contrast, the intestinal CYP2C37 activity of mice treated with low doses of FBOT, TP, EGCG, and caffeine was 3.77, 3.39, 6.39, and 3.56 times that of the control group.

The mRNA expression of CYP3A11 did not differ between the control and CMC groups (Fig. 4b). The level of CYP3A11 mRNA expression in the small intestine was found to be significantly increased by rifampicin, reaching 2.57 times that of the control group. In contrast, ketoconazole induced a reduction in expression, but only by 63% of the control group (*P* < 0.05). The mRNA expression of CYP3A11 was significantly increased in the small intestine of mice administered high and low doses of FBOT, TP, EGCG, and caffeine, compared to the control group (1.94, 1.32, 1.88, 1.30, 2.09, 1.45, 1.73, and 1.25 times, respectively). Consistent regulation of CYP3A11 enzyme activity and mRNA by treatments was observed. The observation suggested that the CYP3A activity increase induced by the FBOT extract was controlled by the CYP3A11 transcription level, which was primarily associated with the bioactive compounds present in the FBOT extract (TP, EGCG, and caffeine).

### The effects of FBOT extract and its bioactive compounds on ATPase activity in mice intestine

**Fig. 5:**
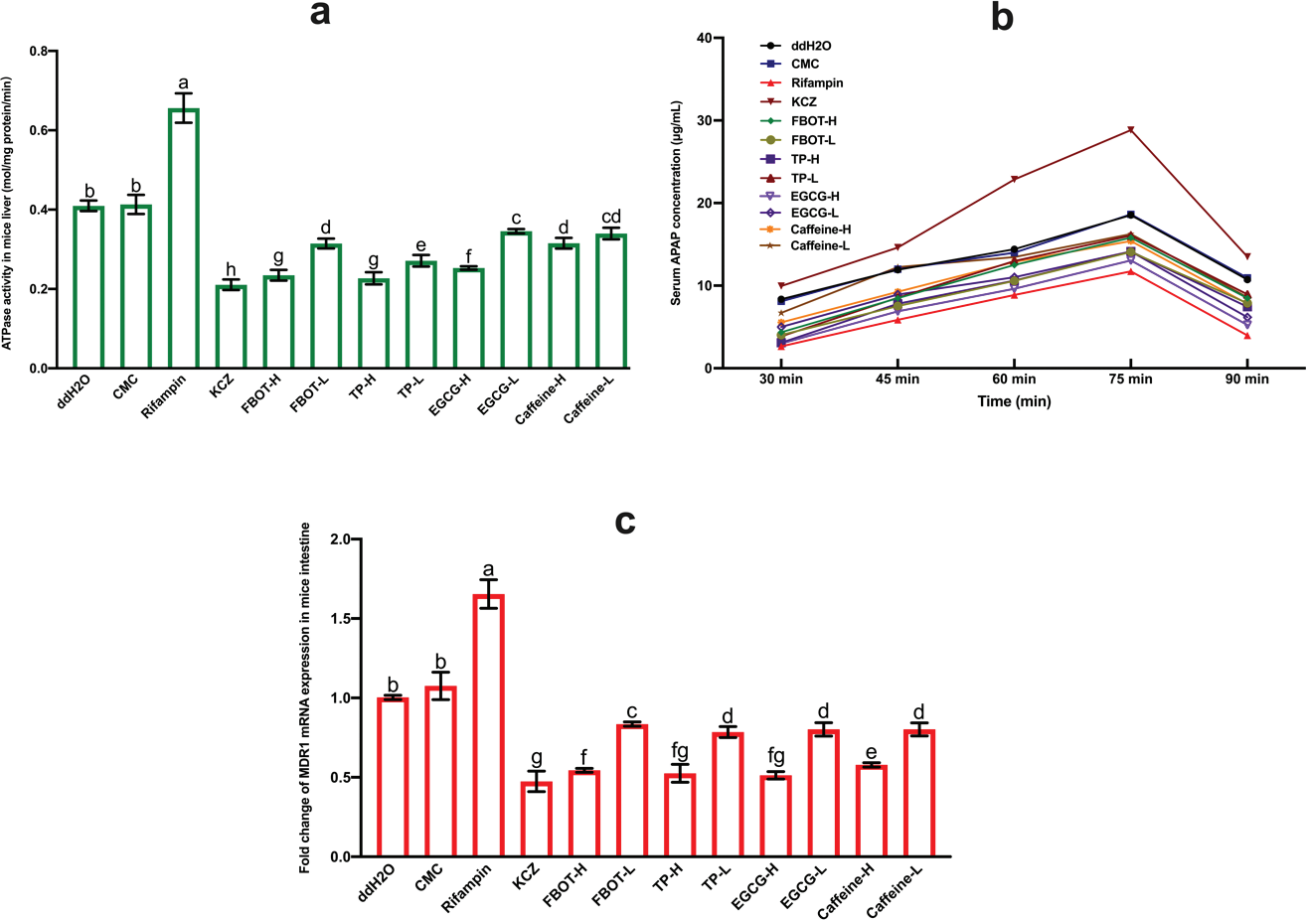
The ATPase activity in mice liver (a), serum APAP concentration in mice (b) and MDR1 mRNA expression (c). Values are mean ± SD (*n* = 3). Bars with different letters represent a significant difference between each group (*P* < 0.05). FBOT-L and FBOT-H mean low and high dose of freeze-drying Baiyedancong Oolong tea, respectively; TP-H and TP-L mean high and low dose of tea polyphenols in BOT, respectively; EGCG-H and EGCG-L mean high and low dose of Epigallocatechin-3-gallate (EGCG) in BOT, respectively; caffeine-H and caffeine-L mean high and low dose of caffeine in BOT, respectively.

There are currently no reported direct indicators for detecting P-gp function *in vivo*. At present, the indicator of P-gp function is frequently the ATPase activity or drug accumulative concentration that drives P-gp. As illustrated in Fig. 5a, the P-gp coupled ATPase activity in the intestine of mice treated with CMC did not differ significantly from that of the control group. However, rifampicin generated a 1.60-fold increase in ATPase activity in the mice’s intestine, which was statistically significant (*P* < 0.05). Furthermore, the ATPase activity was significantly diminished by ketoconazole, decreased by 0.51 times compared to the control group (*P* < 0.05). The ATPase activities of the control group were enhanced by a mere 47, 68, 55, 66, 61, 84, 77, and 82%, respectively, when high and low doses of BOT, TP, EGCG, and caffeine were administered. ATPase, a crucial enzyme that supplies energy to P-gp, was found to be significantly inhibited in the intestines of mice in response to all treatments. This compromised the transport function of P-gp in the intestine, leading to a decrease in the number of substrate drugs absorbed into the bloodstream and an increase in the quantity absorbed via the intestine.

### The effects of FBOT extract and its bioactive compounds on mice serum APAP concentration

The multipoint serum concentration of APAP, a typical P-gp substrate drug, was utilized to validate ATPase activity and deduce whether the transport capacity of P-gp was regulated. The concentration of serum APAP was determined at five time points 1 h after the final gavage: 30, 45, 60, 75, and 90 min. The results are visually represented in Fig. 5b. Typically, the peak concentration of APAP was observed at the 75-min mark. The absence of a statistically significant distinction (*P* > 0.05) between the control and CMC groups indicated that the administration of CMC did not have a substantial impact on the serum APAP concentration of mice. Ketoconazole, a P-gp inhibitor, significantly increased serum APAP concentration, suggesting that it enhanced intestinal APAP absorption and reduced the efflux effect of P-gp on substrate APAP. Furthermore, it was apparent that the potent inducer rifampicin resulted in a reduction of the serum APAP concentration in mice. This finding indicated that rifampicin could reduce the intestinal absorption of the substrate APAP by enhancing the efflux effect of the transporter P-gp for the substrate APAP. In addition, the APAP concentration in mice that received BOT, TP, EGCG, and caffeine was found to be higher than that of the control group, but comparatively lower than that of the group that received ketoconazole.

The mRNA expression of small intestine MDR1 in mice was quantified using an RT-PCR assay in order to determine whether the decreased transport capacity of P-gp was due to weakened transcription of the gene encoding MDR1. The illustrations of the outcomes are presented in Fig. 5c. The pattern of MDR1 mRNA expression was comparable to that of serum APAP concentration. The bioactive compounds of ketoconazole and FBOT exhibited a significant reduction in the expression of MDR1 mRNA in the small intestine when compared to the control group (*P* < 0.05). In conjunction with serum APAP concentration analysis and mRNA expression, the regulation of MDR1 mRNA expression and P-gp capacity was consistent; FBOT extract and its bioactive compounds significantly inhibited these processes. This discovery suggested that the diminished P-gp transport capability induced by FBOT extract was predominantly governed by the downregulation of MDR1 transcription level and was significantly associated with the caffeine, EGCG, and TP present in FBOT.

### Effects of FBOT extract and its bioactive compounds on PAH concentration in renal sections, renal OAT1 and OAT3 mRNA expressions in mice

**Fig. 6:**
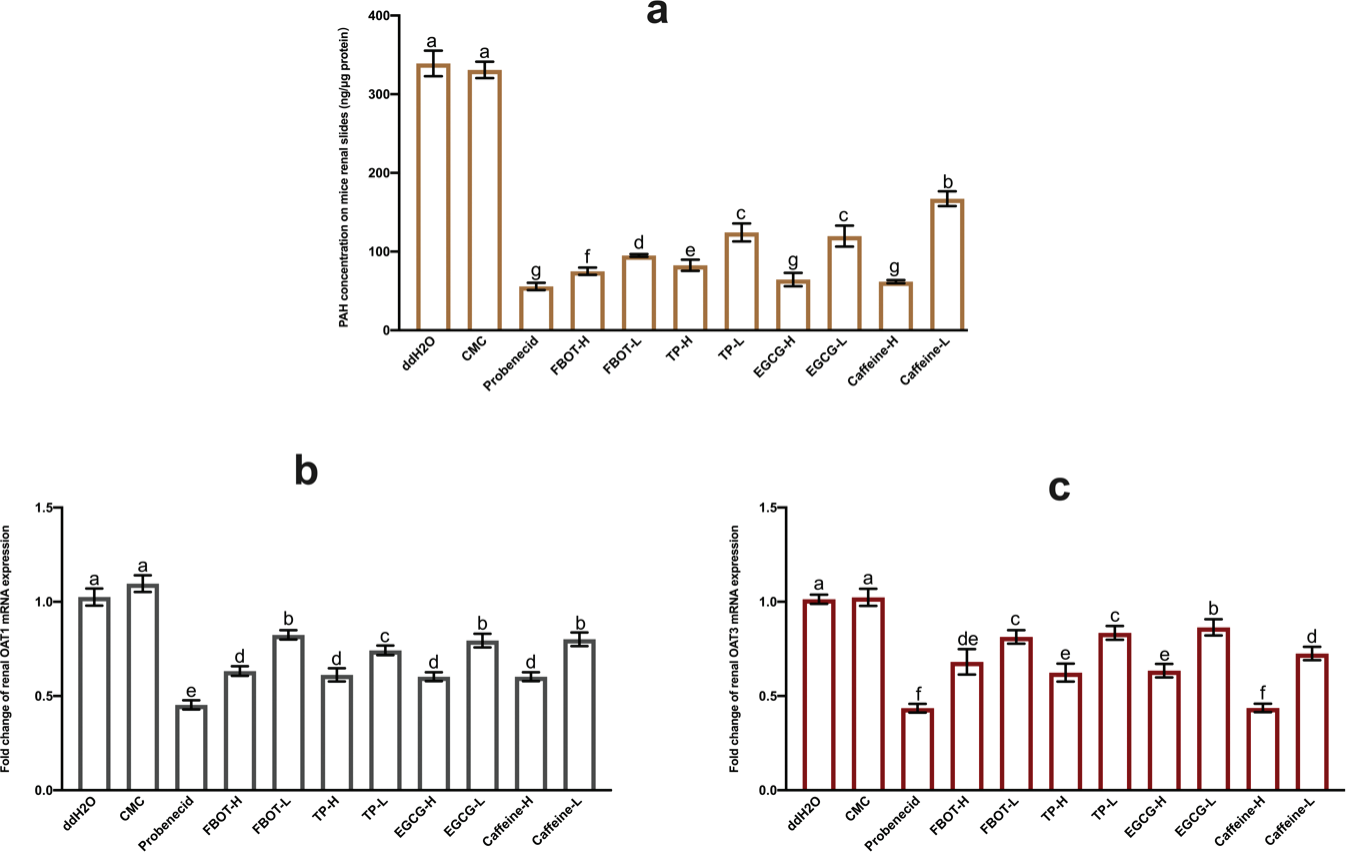
The PAH concentration on mice renal slides (a), OAT1 (b) and OAT3 mRNA expressions (c) in mice kidney. Values are mean ± SD (*n* = 3). Bars with different letters represent a significant difference between each group (*P* < 0.05). FBOT-L and FBOT-H mean low and high dose of freeze-drying Baiyedancong Oolong tea, respectively; TP-H and TP-L mean high and low dose of tea polyphenols in BOT, respectively; EGCG-H and EGCG-L mean high and low dose of Epigallocatechin-3-gallate (EGCG) in BOT, respectively; caffeine-H and caffeine-L mean high and low dose of caffeine in BOT, respectively.

PAH can be transported almost exclusively by renal OATs at low concentrations. The impact of aqueous extracts from BOT on the transport capability of OATs was validated in this study through the quantification of PAH uptake in kidney sections through *in vitro* incubation. Figure 6a illustrates the results.

The fact that the concentration of PAHs did not differ significantly between the control and CMC groups (*P* > 0.05) suggested that CMC had little effect on the ability of OATs to absorb PAH. Probenecid, a potent inhibitor of transporter OATs, significantly reduced the concentration of PAH in renal sections compared to the control group (*P* < 0.05). The concentration was decreased to 16% of the control group’s level. Similarly, PAH concentrations in renal sections were decreased in a dosage-dependent manner by caffeine, FBOT, TP, and EGCG. The treatment groups for FBOT, TP, EGCG, and caffeine were as follows: 25, 33, 24, 36, 19, 35, 18, and 49%, respectively, of the control group. Renal OATs are crucial in the process of organic anion elimination from the body, which includes the elimination of uric acid. Based on these findings, it appears that BOT extract and its bioactive compounds have the potential to impede the transport capability of OATs, consequently leading to a decrease in the renal excretion of substrate drugs or drug metabolites.

The mRNA expressions of renal OAT1 and OAT3 in mice were analyzed to determine whether decreased transcription levels of OAT1 and OAT3 are the cause of the decreased OAT transport capacity. The findings are illustrated in Fig. 6b and c. In general, the levels of OAT1 and OAT3 mRNA exhibited comparable trends in relation to the concentration of PAH. Caffeine, TP, EGCG, probenecid and FBOT extract, and TP all significantly inhibited the expression of OAT1 and OAT3.

Moreover, high-dose caffeine showed strong inhibition on OAT3 mRNA expression, with a relative expression level of 44.87% of the control group. Combined with the concentration of PAH in renal sections, the PAH concentration in the caffeine-H group was also extremely significantly reduced, and the reduction effect was similar to that in the treatment of probenecid. The inhibition of high-dose caffeine on OAT3 mRNA expression was better matched with the inhibition of PAH uptake by OATs when compared with OAT1 mRNA expression.

## Discussion

Oolong tea is a semi-fermented tea and is rich in polyphenols and its oxidizing polymers, alkaloids, and amino acids (23). The effect of Oolong tea on drug metabolisms may be different from that of black and green tea. As an important representative of Oolong tea in China, BOT was approved as a national tea tree in 2002 by the Crop Variety Certification Committee of the Ministry of Agriculture. It has obvious unique properties in both endoplasmic composition and sensory characteristics.

Studies have indicated that tea polyphenols, especially EGCG, exhibit varying anticancer effects in different organs, which might be different from the effective concentration in each organ (24). As a result, the effects of EGCG on drug-metabolizing enzymes and transporters may vary. Although the biological availability of EGCG is not high, it is easy to accumulate in the intestinal tract, liver, and lung and may obtain effective biological concentration, thus affecting the drug metabolism process through altering the activities of a variety of CYP450 isoenzymes and transporters, while the biological availability of caffeine can reach almost 100%, which can be quickly and completely absorbed by the human system and pass through various barriers such as blood–brain barrier (25). Its effects on drug-metabolizing enzymes and transporters are likely to be systemic. Caffeine is a metabolizing substrate drug for CYP1A2, which can significantly reduce the activity of CYP1A2 and also affect other CYP450 isoenzymes (26). Therefore, this study compared the effects of aqueous extracts from FBOT, and its bioactive compounds of TP, EGCG, and caffeine on drug metabolisms to verify whether the EGCG and caffeine are the target components in BOT extract that can regulate the mRNA expression as well as its corresponding activities of liver CYP3A, CYP2E1 and CYP2C37, small intestine CYP3A, and P-gp and OATs transport capacities. The results indicated that the enzyme activities of CYP3A, CYP2E1, and CYP2C37 in the liver and CYP3A in the small intestine of mice were significantly increased in both high- and low-dose treatment groups, while the transport capacities of P-gp and OATs were significantly decreased. Moreover, EGCG and caffeine are the main bioactive components in BOT extract that could regulate these metabolic enzymes and transporters. EGCG-H treated liver CYP3A11, CYP2C37, and small intestine CYP3A11 mRNA expressions were significantly higher than that of the caffeine-H treated group, while the effect of caffein-H on the inhibition of OAT3 mRNA expression was significantly stronger than that of the EGCG group. Studies on the regulatory mechanism of CYP450 have shown that transcriptional and translational levels and post-translational modifications may have vital effects on the activities of metabolic enzymes (8).

Aqueous extract from FBOT and its tea polyphenols, EGCG, and caffeine significantly regulated the transcriptional levels of CYP450 metabolic enzymes and transporters in mice, including inducing the transcriptional levels of CYP3A11, CYP2C37, and CYP3A11 in liver and small intestine. However, there was significant inhibition of liver CYP2E1, MDR1 encoding the effluence transporter P-gp, and OAT1 and OAT3 transcription levels. These results indicated that these regulatory abilities were strongly related to the tea polyphenols, EGCG, and caffeine in the FBOT extract. However, the regulation of transcription levels of CYP3A11, CYP2C37, and CYP2E1 by FBOT extracts was not the sum of the regulation of their individual components (EGCG and caffeine), and each component was likely to be competitive in regulation. Studies have shown that the transporter P-gp could be regulated by the activation of PXR by ligands such as rifampicin and St. John’s grass extract containing flavonoids (27). Herein, whether EGCG and caffeine regulate P-gp through this pathway needs further exploration.

Compared with EGCG and caffeine treatment groups, the FBOT treatment group containing equal amounts of EGCG and caffeine did not significantly increase the regulation of enzyme activities and transporter capacities, and the induction of liver CYP3A, CYP2E1, and CYP2C37, and small intestine CYP3A activities was weakened than that of the EGCG treatment group. These results suggested that EGCG and caffeine did not induce CYP450 and inhibit the transporter activities in a cumulative manner, but in a competitive manner. Furthermore, at the same dose level, the induction of EGCG and caffeine on liver CYP3A, CYP2E1, and CYP2C37, and small intestine CYP3A activities was significantly different. The effects of EGCG on serum APAP and OATs were also different from that of caffeine at low-dose levels. Hence, it can be speculated that different substances in the BOT extract may have different regulatory effects on the same CYP450 metabolic enzyme or transporter. Besides, the effects of other bioactive components such as theanine in the extract on CYP450 metabolic enzymes and transporters need to be further studied.

The difference in the concentration and the dosage of bioactive components in tea can affect the expression of metabolic enzymes and transporters *in vivo*. In summarizing the effects of green tea polyphenols on drug metabolism, Chung, Choi (28) proposed that the dosage of tea extracts used for specific functions was too high, such as the consumption of green tea extract capsules containing 1,000 mg of EGCG twice a day, which was equal to the same amount as three cups of 2.5 g of green tea in one sitting. This may have a significant impact on drug metabolism in the body. In this study, the significant differences in the expression of CYP450 isoenzyme and transporter by high-dose and low-dose of FBOT extracts, TP, EGCG, and caffeine also verified the inference of previous study, which could have a promising reference for the safe consumption of tea during the drug administration.

The high- and low-dose treatments used in this study simulated the situation of daily tea consumption as well as the high-dose use of tea extract. However, only the simulation of these two doses is far from enough to demonstrate the potential effects of tea consumption on drug metabolisms. Such studies as more varying dosages of tea consumption on drug metabolisms and transporters in various organs and their underlying mechanisms should be investigated.

## Conclusion

Aqueous extracts derived from FBOT and its bioactive compounds (TP, EGCG, and caffeine) were found to enhance the activities of liver CYP2E1, CYP2C37, and small intestinal CYP3A in mice. Conversely, these extracts were found to reduce the transport capacity of renal ingestion transporter OATs and intestinal effluent transporter P-gp. In addition to the liver CYP2E1 activity, these activities may be regulated by the transcription levels of their corresponding encoding genes. Therefore, it significantly impacted the hepatic metabolism, intestinal absorption, and renal excretion of oral drugs. However, the final concentration of EGCG and caffeine in the liver and small intestine of mice was not determined, which is also the deficiency of this study. Comprehensive consideration should be taken in subsequent studies, including studies on CYP450 enzyme activity and transporters in other organs to better evaluate the potential influence of BOT on drugs and provide a basis for tea consumption during drug administration scientifically. Besides, neither animal nor cell experiments can serve as substitutes for clinical trials, which will be the focus of our forthcoming research in order to validate the impact of tea on the effects of pharmaceuticals.
